# Genetic Contribution of Femoral Neck Bone Geometry to the Risk of Developing Osteoporosis: A Family-Based Study

**DOI:** 10.1371/journal.pone.0154833

**Published:** 2016-05-10

**Authors:** Nerea Hernandez-de Sosa, Georgios Athanasiadis, Jorge Malouf, Ana Laiz, Ana Marin, Silvia Herrera, Jordi Farrerons, Jose Manuel Soria, Jordi Casademont

**Affiliations:** 1 Department of Internal Medicine, Hospital de la Santa Creu i Sant Pau, Universitat Autònoma de Barcelona, Barcelona, Spain; 2 Department of Genomics of Complex Diseases, Research Institute, Hospital de la Santa Creu i Sant Pau, Barcelona, Spain; 3 Bioinformatics Research Centre, Aarhus University, Aarhus, Denmark; Charles P. Darby Children's Research Institute, 173 Ashley Avenue, Charleston, SC 29425, USA, UNITED STATES

## Abstract

Femoral neck geometry parameters are believed to be as good as bone mineral density as independent factors in predicting hip fracture risk. This study was conducted to analyze the roles of genetic and environmental factors in femoral properties measured in a sample of Spanish families with osteoporotic fractures and extended genealogy. The “Genetic Analysis of Osteoporosis (GAO) Project” involved 11 extended families with a total number of 376 individuals. We studied three categorical phenotypes of particular clinical interest and we used a *Hip structural analysis* based on DXA to analyze 17 strength and geometrical phenotypes of the hip. All the femoral properties had highly significant heritability, ranging from 0.252 to 0.586. The most significant correlations were observed at the genetic level (ρ_G_). Osteoporotic fracture status (Affected 2) and, particularly, low bone mass and osteoporotic condition (Affected 3) had the highest number of significant genetic correlations with diverse femoral properties. In conclusion, our findings suggest that a relatively simple and easy to use method based on DXA studies can provide useful data on properties of the Hip in clinical practice. Furthermore, our results provide a strong motivation for further studies in order to improve the understanding of the pathophysiological mechanism underlying bone architecture and the genetics of osteoporosis.

## Introduction

Hip fracture is recognized as the most serious osteoporotic fracture due to its association with increased morbidity and mortality, and decreased functional capacity with a one-year mortality of approximately 20% [1, 2]. It is therefore important to quantify the assorted genetic and environmental factors that may contribute to osteoporotic fractures in order to help to prevent hip fracture in particular.

A standard method of measuring proximal femur strength is bone biopsy. However, because biopsy is to invasive to be used in routine clinical practice, a series of alternative methods have been developed. By far, the most common method is measuring bone mineral density (BMD) at different skeletal parts, as this correlates sufficiently with the risk of developing osteoporotic fractures [3–6].

Femoral neck geometry parameters (FNGPs) that measure bone structural properties such as shape and size are believed to be as good independent factors in predicting hip fracture risk as BMD is [7]. There is abundant evidence suggesting a genetic contribution to several of such FNGP indices [8, 9], with heritability estimates (*h*^*2*^) ranging from 0.37 to 0.62 [10–12]. In addition, different loci affecting FNGPs have been identified by diverse linkage [13–19] and genome-wide association studies (GWAS) [11–12, 18, 20].

The best non-invasive approach to determine FNGPs requires imaging technologies and computational methods such as using quantitative computed tomography that are prohibitively expensive or require an unacceptably high radiation dose [21, 22]. In recent years, hip structural analysis (HSA) based on dual X-ray absorptiometry (DXA) has become available. Although the analysis is restricted to just two dimensions and the resolution of structural dimensions is admittedly low [23], HSA seems to be an acceptable approach to analyze strength and geometrical properties of the hip in the clinical setting with the additional advantages of (i) a relatively low cost and (ii) a small radiation dose compared to quantitative computed tomography [24, 25].

In this study, we aim to analyze the relative importance of genetic and environmental factors in FNGPs measured by means of DXA-based HSA in a set of extended Spanish families characterized by osteoporotic fractures. One of the strengths of our study is precisely its extended design, which provides reliable estimates for the genetic contribution to the studied phenotypes. Unlike twin studies, family-based designs leverage different degrees of kinship allowing for a smaller sampling variance. However, at the same time, the inclusion of many members of the same family makes the separation of genetic from common environmental effects more challenging from a computational perspective [26].

## Materials and Methods

**Fig 1:**
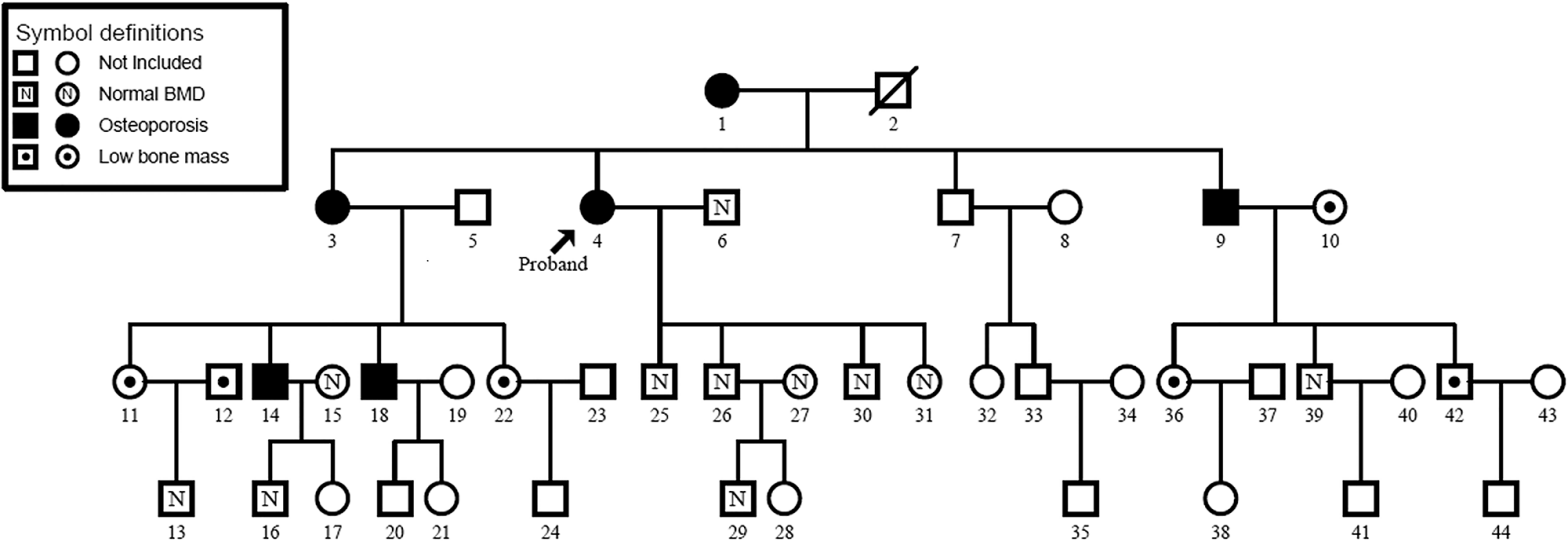
GAO project pedigree sample (Family Nr. 3).

The patients included in the analysis are from the Genetic Analysis of Osteoporosis (GAO) Project. The GAO Project is a genetic study based on extended pedigrees from Spain and the selection, recruitment and methodology have been described in detail elsewhere [27, 28]. In brief, the project recruited 11 extended families from Barcelona, Spain, between March 2009 and March 2012. All families were selected through a proband with osteoporosis and a family had to have at least ten living individuals distributed in three or more generations. The structure of the families was verified by use of microsatellite genotyping and control for Mendelian inconsistencies with FBAT v2.0.3 [29]. The GAO project pedigree sample shown in Fig 1. Adult subjects gave informed consent for themselves and for their underage family members. The Ethical committee of Clinical Investigation of Hospital de la Santa Creu i San Pau approved all recruitment protocols (08/015/281).

A medical history was obtained from all the participants and it included information about menstrual period, history of all clinical fractures (traumatic and atraumatic) and current medication with a known negative (e.g. corticoids, heparin, proton pump inhibitors, insulin or thiazolidinediones) or positive (e.g. bisphosphonates, calcium, strontium, parathyroid hormone, thiazide diuretics, vitamin D) effect on bone remodeling. Coffee, alcohol and smoking habits, dietary calcium intake, sun exposure and physical activity were also recorded.

**Fig 2:**
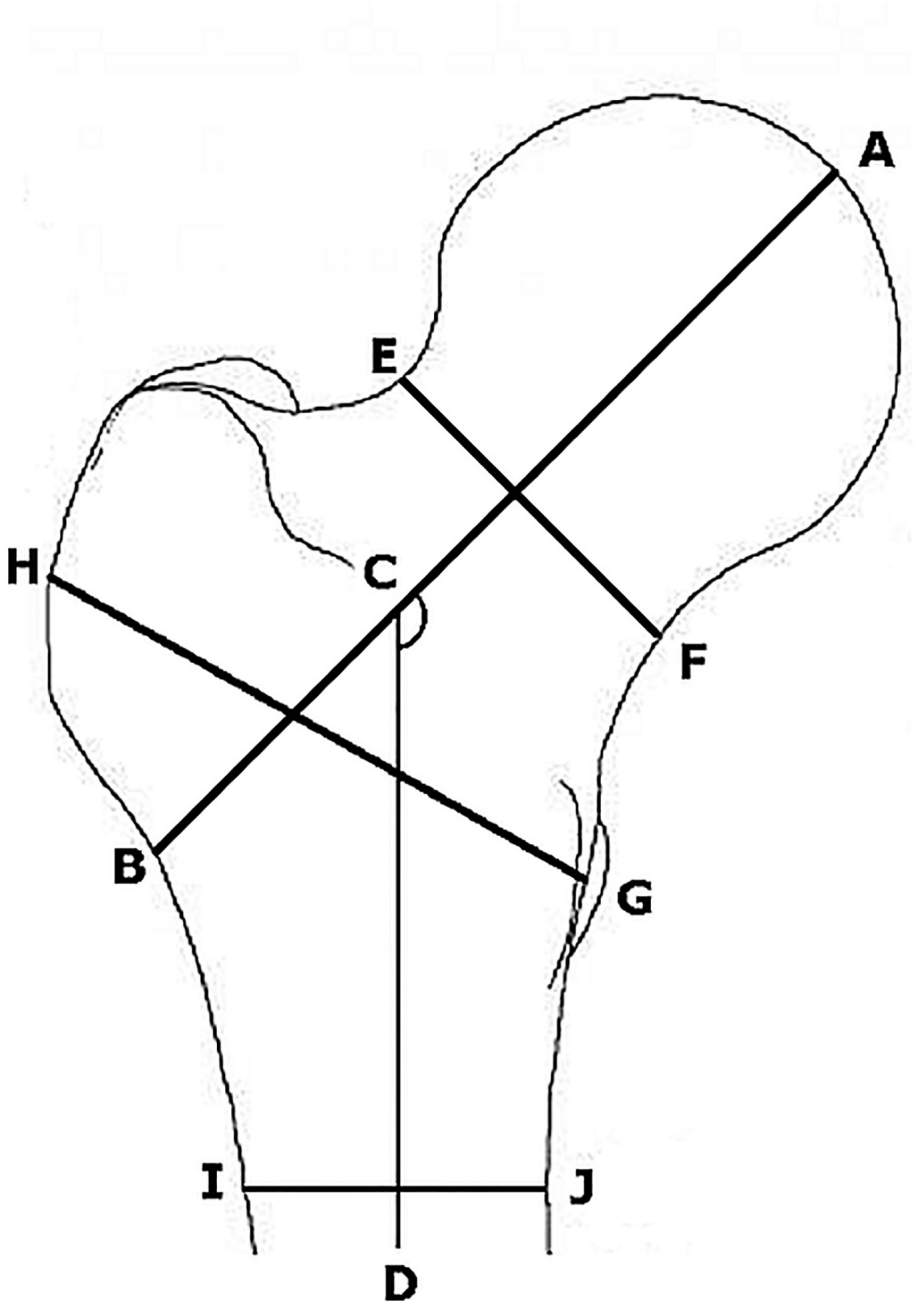
Measurement of the femur geometric parameters. Structural traits: AB is the hip axis length (HAL), ACD is the femoral neck-shaft angle (NSA), EF is the Narrow Neck, HG is the Intertrochanteric and IJ is the Femoral Shaft.

Spine, femur and whole-body DXA scans were performed on all participants using a Discovery dual energy X-ray absorptiometry (DXA) system with the APEX v2.3 software (Hologic, Bedford, MA, USA), following the manufacturer’s recommendations and analyzed by one expert technician. We used the HSA software to analyze strength and geometrical properties of the hip [30]. This program uses the distribution of mineral mass in a line of pixels across the bone to measure geometric properties of cross-sections in cut planes traversing the bone at that location [31]. Three regions were analyzed: (i) narrow neck (NN), across the narrowest diameter of the femoral neck; (ii) intertrochanteric (IT), along the bisector of the neck-shaft angle; and (iii) femoral shaft (FS), 2 cm distal to the midpoint of the lesser trochanter (Fig 2). For each region, the distribution of the bone mass across the bone was extracted and geometric properties were derived using diverse formulas described elsewhere [31]. The FNGP variables considered were the following: buckling ratio (BR), an index of bone structural instability indicating the risk of fracture by buckling; cross-sectional area (CSA), an indicator of bone axial compression strength; cortical thickness (CT), an indicator of mean cortical thickness; cross-sectional moment of inertia (CSMI), an index of structural rigidity; and section modulus (Z), an index of bone bending strength indicating the bending resistance of a tube.

Our study focused on 17 structural phenotypes that we considered of high clinical relevance. Table 1 contains a description of the phenotypes as well as a guide for the abbreviations used. We also studied three categorical osteoporotic phenotypes of particular interest: “Affected 1” corresponds to individuals ≥ 21 years-old resenting one or more of the following characteristics: (i) T-score < -2.5 (spine, hip neck or total hip); (ii) at least one osteoporotic (atraumatic) fracture; (iii) antiresorptive or forming agent treatment; “Affected 2” corresponds to patients suffering at least one osteoporotic fracture; and “Affected 3” corresponds to a broad category of low bone mass and osteoporotic patients, i.e. encompassing individuals classified as “Affected 1” as well as individuals with a T-score < -1 (spine, hip neck or total hip). Definitions of categorical phenotypes were described in detail elsewhere [27].

**Table 1:** Description of the phenotypes studied in the GAO Project.

	Trait abbreviation	Description
**Structural traits**	HAL	Hip axis length (mm)
	NSA	Femoral neck—shaft angle (degrees)
**Strength properties**	FS-CT	Average cortical thickness of femoral shaft (cm)
	FS-BR	Buckling ratio of femoral shaft (cm^3^)
	FS-CSA	Cross-sectional area of femoral shaft (cm^2^)
	FS-CSMI	Cross-sectional moment of inertia of femoral shaft (cm^4^)
	FS-Z	Section modulus of femoral shaft (cm^3^)
	IT-CT	Intertrocanteric average cortical thickness (cm)
	IT-BR	Intertrocanteric buckling ratio (cm^3^)
	IT-CSA	Intertrocanteric Cross-sectional area (cm^2^)
	IT-CSMI	Intertrocanteric Cross-sectional moment of inertia (cm^4^)
	IT-Z	Intertrocanteric section modulus (cm^3^)
	NN-CT	Average cortical thickness of narrow neck (cm)
	NN-BR	Buckling ratio of narrow neck (cm^3^)
	NN-CSA	Cross-sectional area of narrow neck (cm^2^)
	NN-CSMI	Cross-sectional moment of inertia of narrow neck (cm^4^)
	NN-Z	Section modulus of narrow neck (cm^3^)

*HAL (mm)*: the distance from pelvic rim to outer margin of greater trochanther along neck axis. *NSA (degrees)*: angle between derived axes of neck and shaft. *FS*: the femoral shaft, *CT (cm)*: estimate of mean cortical thickness. *BR (cm*^*3*^*)*: Relative thickness of the cortex as an estimate of cortical stability in buckling. *CSA (cm*^*2*^*)*: equivalent to the amount of (cortical equivalent) bone surface area in the cross-section after excluding all trabecular and soft tissue spaces. *CSMI (cm*^*4*^*)*: for bending in the image plane from bone mass profile integral. Index of structural rigidity; reflects distribution of mass about the center of a structural element. *Z (cm*^*3*^*)*: Indicator of bending strength for maximum bending stress in the image plane. *IT*: Intertrochanteric. *NN*: Narrow neck. [30]

Statistical analysis was based on a variance component analytical framework in which maximum likelihood techniques were used to assess *h*^*2*^, as well as genetic and environmental correlations (ρ_G_ and ρ_E_) among the studied phenotypes. Any significant correlation ≥ 0.70 was considered strong and of clinical interest.

In more detail, we used a mixed linear model to determine the contribution of genetic and individual-specific environmental factors to the variation of the quantitative and the categorical osteoporotic phenotypes. We modeled the measurement of a trait y for individual i (y_i_) as a linear function using the following formula:

yi=μ+∑βjΧij+gi+ei

Whereby μ is the trait’s mean, x_ij_ is the j^th^ covariate and β_j_ is its regression coefficient.

Covariates included age, age^2^, gender, body mass index (BMI), age of menopause for post-menopausal women, alcohol intake, smoking status and use of osteoporosis-related medication, as well as interactions of age and age^2^ with gender. The remaining variables, g_i_, and e_i_, represent the random deviations from μ for individual i that are attributable to additive genetic and residual non-genetic effects, respectively. The effects of g_i_, and e_i_ were assumed to be independent and normally distributed with mean = 0 and variances σ_g_^2^ and σ_e_^2^.

The statistical software SOLAR v4.3.1 [32] was used to estimate simultaneously the mean and variances, as well as the covariate and genetic effects for each trait. We assessed the significance of such effects with a likelihood-ratio test. [33, 34] Finally, we estimated the heritability for each trait as the proportion of the total phenotypic variability attributable to additive genetic effects. For this particular estimation, we considered only environmental covariates (i.e. we performed the analysis without BMI or age of menopause for post-menopausal women).

To study the genetic relationships between categorical and quantitative phenotypes, we used a modified variance component method for mixed discrete/continuous traits [35] incorporated in SOLAR. This method allowed for the phenotypic correlations between pairs of traits to be separated into common genetic and common environmental influences. As above, the environmental component includes all non-genetic risk factors that may be influencing the phenotype after excluding covariate effects (sun exposure, diet, habits, medical treatment, sex, age and BMI).

## Results

Summary statistics of interest for the studied pedigrees, as well as a description of the probands used in the recruitment have been described elsewhere [27]. In brief, the sample included 367 individuals with sample size per family ranging from 15 to 91 (mean = 33; median = 30). Age ranged from 5 to 93 (mean = 40.8; median = 41) and male: female ratio of 1.07. Approximately 32.1% of women and 44% of men were smokers, 31.6% of women were postmenopausal and 3.2% of the entire sample was diabetics. The general characteristics of the patients in categorical phenotypes were described in Table 2. The number of osteoporotic patients (Affected 1) was 70. Twenty-four individuals had osteoporotic fractures (Affected 2 patients) representing the 6.5% of the total sample size. Three probands presented multiple osteoporotic fractures. In nine out of 11 pedigrees, there were one or two patients with vertebral or femoral non-traumatic fractures. Finally, patients with low bone mass, corresponding to Affected 3 group of patients, were 206.

**Table 2:** Description of the general characteristics of the patients in each of the three categorical phenotypes.

Categorical phenotype	N	Median Age	M:F ratio	Median BMI	Median Age menopause	Median T-score		
						Femoral neck BMD	Hip BMD	Total spine BMD
**Affected 1**	70	61.5	0.49	25.74	49	-2.2	-1.35	-2.85
**Affected 2**	24	65	0.41	24.97	50	-1.65	-0.80	-1.60
**Affected 3**	206	49.5	0.75	24.91	49	-1.50	-0.75	-1.80

Affected 1: osteoporotic patients; Affected 2: patients with osteoporotic fractures; Affected 3: patients with low bone mass; N: number size; M: male; F: female.

Heritability of each of the structural phenotypes is shown in Table 3. All the femoral properties showed statistically significant heritability, ranging from 0.252 (FS-CSA) to 0.586 (NN-BR) after correcting for covariate effects. The covariates that had a significant effect on femoral properties (p<0.05) appear in Table 4. From the covariates that were initially included in the model, smoking, use of osteoporosis-related medication, alcohol consumption, smoking habits, dietary calcium intake, sun exposure and physical activity did not have any significant effect on the final phenotypes and, therefore, are not shown. Table 4 also shows the proportion of variance of femoral properties that is attributable to such covariates, ranging from 7% (for NSA) to 73% (for FS-Z).

**Table 3:** Heritability of the phenotypes in the GAO Project.

	Trait	h^2^ (h^2^ s)	P value
**Structural traits**	HAL	0.377 (0.081)	2.95 × 10^−10^
	NSA	0.456 (0.110)	3.26 × 10^−08^
**Strength properties**	FS-CT	0.394 (0.098)	2.5 × 10^−06^
	FS-BR	0.454 (0.090)	1.22 × 10^−09^
	FS-CSA	0.252 (0.094)	8.84 × 10^−04^
	FS-CSMI	0.430 (0.098)	2 × 10^−07^
	FS-Z	0.354 (0.098)	9.6 × 10^−06^
	IT-CT	0.492 (0.096)	1.77 × 10^−08^
	IT-BR	0.493 (0.083)	5.61 × 10^−12^
	IT-CSA	0.353 (0.098)	1.5 × 10^−05^
	IT-CSMI	0.314 (0.098)	5.72 × 10^−05^
	IT-Z	0.328 (0.104)	1.25 × 10^−04^
	NN-CT	0.472 (0.104)	5 × 10^−07^
	NN-BR	0.586 (0.087)	1.46 × 10^−12^
	NN-CSA	0.308 (0.099)	2.44 × 10^−04^
	NN-CSMI	0.386 (0.097)	3.9 × 10^−06^
	NN-Z	0.277 (0.094)	4.6 × 10^−04^

*(h*^*2*^ *s)*: *h*^*2*^ *standard error*. See Table 1 for acronym descriptions.

**Table 4:** Regression coefficients for statistically significant covariate effects.

	Trait	Age	Female gender	BMI	Menopause age	Var. expl.
**Structural traits**	HAL	0.216	-1.5.9			0.558
	NSA		-1.801		0.052	0.0754
**Strength properties**	FS-CT		-0.07	0.009	-0.002	0.3557
	FS-BR	0.008		-0.024	0.007	0.196
	FS-CSA	0.016	-1.077	0.081	-0.008	0.6889
	FS-CSMI	0.027	-1.662	0.1		0.697
	FS-Z	0.014	-0.823	0.054		0.7309
	IT-CT		-0.035	0.004	-0.001	0.2362
	IT-BR	0.047		-0.065	0.016	0.2908
	IT-CSA	0.01	-1.057	0.074	-0.011	0.4811
	IT-CSMI	0.093	-5.499	0.298		0.6109
	IT-Z	0.02	-1.328	0.089		0.5594
	NN-CT	0		0.002	0	0.199
	NN-BR	0.074	-1.141	-0.08		0.2804
	NN-CSA		-0.533	0.044	-0.006	0.4325
	NN-CSMI	0.015	-1.399	0.063		0.5732
	NN-Z	0.005	-0.524	0.026	-0.004	0.508

Only significant p values are shown (p<0.05). Empty cells denote a non-significant effect (p ≥ 0.05). Var. expl.: variance explained by adjusted covariates; see Table 1 for acronym descriptions.

Table 5 shows the correlation of each of the 17 structural phenotypes with the three categorical phenotypes of clinical interest on the genetic (ρ_G_) and environmental (ρ_E_) level. The majority of the correlations were negative. This is in accord with the expected negative correlation between osteoporotic phenotypes and cortical thickness (CT), cross-sectional areas (CSA), cross-sectional moment of inertia (CSMI) and section modules ((Z). On the contrary, the correlations were in general positive, as expected, between osteoporotic phenotypes and buckling rations (BR), a measure of cortical instability in buckling. Supporting information contains the full list of correlations among all combinations of phenotypes on the phenotypic, genetic and environmental level for the interested reader S1 File.

**Table 5:** Genetic and environmental correlation of intermediate phenotypes based on femoral geometry parameters with three different osteoporotic phenotypes.

	Trait	Affected1 n = 70		Affected2 n = 24		Affected3 n = 206	
		ρ_G_	ρ_E_	ρ_G_	ρ_E_	ρ_G_	ρ_E_
**Structural traits**	**HAL**	-0.208	0.003	0.234	-0.098	0.010	0.241
	**NSA**	-0.122	-0.055	-0.023	-0.302	0.154	-0.267
**Strength properties**	**FS-CT**	-0.543	-0.354	-0.484	-0.139	-0.781*	0.149
	**FS-BR**	0.488	0.460	0.770*	-0.011	0.644	0.179
	**FS-CSA**	-0.429	-0.082	0.473	-0.230	-0.762*	0.320
	**FS-CSMI**	-0.140	0.163	0.896*	-0.259	-0.216	0.379
	**FS-Z**	-0.167	0.108	0.957*	-0.271	-0.281	0.396
	**IT-CT**	-0.758*	-0.452	-0.515	-0.024	-0.954*	-0.107
	**IT-BR**	0.824*	0.509	1.000*	-0.027	0.775*	0.732*
	**IT-CSA**	-0.641	-0.161	-0.076	-0.122	-0.857*	0.160
	**IT-CSMI**	-0.537	0.110	0.395	-0.091	-0.669	0.340
	**IT-Z**	-0.634	0.058	0.244	-0.116	-0.751*	0.275
	**NN-CT**	-0.497	-0.693	-0.178	-0.390	-0.617	-0.440
	**NN-BR**	0.458	0.656	0.557	0.161	0.505	0.847*
	**NN-CSA**	-0.526	-0.360	0.282	-0.372	-0.743*	-0.024
	**NN-CSMI**	-0.701*	0.022	0.622	-0.180	-0.748*	0.250
	**NN-Z**	-0.656	-0.124	0.464	-0.253	-0.694	0.089

*: results with relevant genetic correlations.

ρ_G_: genetic contribution; ρ_E_: environmental contribution. See text for the definition on Affected1 to 3. See Table 1 for acronym descriptions.

None of the two structural traits (HAL and NSA) showed strong correlation with any of the three osteoporotic phenotypes analyzed. Regarding the femoral properties representing intermediate structural phenotypes, the most significant correlations were observed on the genetic level (ρ_G_). The highest genetic correlations were found between IT-BR and all different osteoporotic phenotypes (Affected 1–3), ranging from 0.775 to 1.000. Moreover, IT-CT showed high and significant genetic correlations (from -0.758 to -0.954) with Affected 1 and 3. NN-CSMI had also significant genetic correlations, ranging from -0.701 to -0.748, with Affected 1 and 3. Osteoporotic fracture status (Affected 2) and, particularly, low bone mass and osteoporotic condition (Affected 3) had the highest genetic number of significant correlations with diverse femoral properties. In the latter, the correlations were significant in eight over ten femoral traits. Finally, only two significant correlations were observed on the environmental level (ρ_E_), involving Affected 3 and NN-BR (0.847), as well as IT-BR (0.732).

## Discussion

The aim of this study was to provide additional insight into the relative roles of genetic and environmental factors in a series of continuous femoral neck geometry phenotypes and to uncover to which extent such femoral characteristics contribute to a series of well-defined, clinically relevant osteoporotic disease phenotypes. One of the most important advantages of our study is the extent of familial relationships in the sample and the variety of phenotypic traits analyzed.

Diverse studies in the literature have observed the influence of the FNGPs in predicting hip fracture risk [7]. For example, Lacroix et al. [36] described a significant association between hip fracture risk and femur outer diameter (with a 61% of risk) and average buckling ratio (with a 43% of risk). They concluded that hip geometry parameters, particularly intertrochanteric diameter and buckling ratio, predict incident hip fracture after accounting for clinical risk factors and conventional bone density.

Our study conformed that all femoral neck structural traits and strength properties that we studied have a considerable genetic component as manifested by the relatively high *h*^*2*^ values (Table 3). Heritability was highest for NN-BR (58.6%), IT-BR (49.3%) and FS-BR (45.4%). Our estimates of *h*^*2*^, nonetheless, were generally lower than those reported elsewhere [37, 38], which could be explained by the fact that our study was family-based: it is well known that family-based designs provide more conservative estimates of heritability compared to studies based on twins [37, 39–43] or unrelated individuals [43, 44].

An additionally important finding of our study was that most of the geometry factors analyzed were influenced by age, sex, BMI and age of menopause (Table 4). The most significant regression models involved sex as the most important covariate in the femoral geometric traits analyzed. As expected, the sign of the coefficients were negative, indicating that female gender contributes negatively to CT, CSA, CSMI and Z-score. BR, for which a positive regression coefficient should be expected, was not significant in any case.

BMI had an effect opposite to female sex: it was significant and positive for CT, CSA, CSMI and Z, and negative for BR. This is in agreement with the protective effect of a higher BMI in osteoporosis [45]. The contribution of age and age of menopause was significant yet small for many femoral traits. Finally, the poor contribution of age on heritability estimates was in agreement with results obtained for the BMD phenotypes [27].

A third finding worth discussing is the fact that genetic contribution to most FNGPs is higher than the contribution of environmental parameters and that this is particularly true for Affected 3 (Table 5). As expected, BR correlates positively with affected status, as the higher is the BR the more affected are the patients expected to be. On the contrary, the rest of parameters correlated negatively with affected status.

When Affected 1 was analyzed, the behavior of the correlations tended to be similar, although the number of relevant correlations was lower. We hypothesize that the decrease in the number of correlations > 0.70 does not indicate less contribution of genetics to femoral neck parameters in osteoporosis compared with patients with low bone mass, but a lower power to detect such correlations due to the much lower number of patients in this group (n = 66 vs. n = 206 in Affected 3). A similar explanation is plausible for Affected 2 (patients with an osteoporotic fracture) including as few as 24 subjects.

CT, CSA and CSMI are related with bone axial compression strength and structural rigidity. The association of low values of such parameters with a higher risk of hip fracture has already been described previously in the literature [46, 47]. Also, previous GWAS have reported diverse associations between hip geometry traits and genetic variants [48, 49]. The positive correlation between BR and hip fracture risk has also been described in diverse studies [11, 48, 50]. A GWAS identified a significant association between BR and a polymorphism in the *RTP3* gene [13].

In relation to the contribution of environmental factors, the correlations were only relevant for IT-BR and NN-BR in Affected 3 again the group with the highest number of individuals. From our findings, it seems that environmental factors have, on the whole, much less influence on FNGPs than genetic factors do.

Our study is inevitably not free from limitations, the most important of which being the small number of individuals with fragility fractures (Affected 2). These patients provide the most relevant information for uncovering the factors contributing to osteoporotic fractures. Even though this number is low, it is legitimate to assume that Affected 3 (low bone mass) and Affected 1 (osteoporotic) include patients at risk of developing fractures in the future and, thus, at various stages behind Affected 2.

In our analysis, the mean phenotypic correlation of HAL and NSA with six BMD measurements was 0.48 and -0.05, respectively (calculated from S1 File). Given the moderate correlation between HAL and BMD and the lack of correlation between NSA and BMD, it is possible that more incremental information could be obtained from HAL and NSA for estimating low trauma fracture risk when compared to BMD. This observation has potential predictive value beyond the use of BMD in the clinical setting, warranting further investigation in the future.

In conclusion, our findings point out that a relatively easy-to-use DXA-based method can provide useful insights into the involvement of FNGPs in the clinical and research practice. Furthermore, we contribute with additional evidence on the heritability of various FNGPs and that there exists a strong genetic correlation between FNGPs and osteoporotic disease status. Most importantly, our results provide a strong motivation for further studies using state-of-the-art and well-defined GWAS in order to improve the understanding of the pathophysiological mechanism underlying bone architecture and the genetics of osteoporosis.

## Supporting Information

S1 Table Description of the phenotypes studied in the GAO Project. (DOC)

S2 Table Description of the characteristics of the categorical phenotypes studied for sample enrolment. (DOC)

S3 Table Heritability of the phenotypes in the GAO Project. (DOC)

S4 Table Regression coefficients for statistically significant covariate effects. (DOC)

S5 Table Genetic and environmental correlation of intermediate phenotypes based on femoral geometry parameters with three different osteoporotic phenotypes. (DOC)

S1 File The list of correlations. (XLS)
