# Supplementary material for: Genetic Contribution of Femoral Neck Bone Geometry to the Risk of Developing Osteoporosis: A Family-Based Study
Source: PLoS One. 2016 May 10;11(5):e0154833. doi: 10.1371/journal.pone.0154833 (PMC4862643; doi:10.1371/journal.pone.0154833)
Supplement: S1 Table — (DOC) [file pone.0154833.s001.doc]

**Table 1. Description of the phenotypes studied in the GAO Project**.

|  | **Trait abbreviation** | **Description** |
| --- | --- | --- |
| **Structural traits** | HAL | Hip axis length (mm) |
|  | NSA | Femoral neck - shaft angle (degrees) |
| **Strength properties** | FS-CT | Average cortical thickness of femoral shaft (cm) |
|  | FS-BR | Buckling ratio of femoral shaft (cm^3^) |
|  | FS-CSA | Cross-sectional area of femoral shaft (cm^2^) |
|  | FS-CSMI | Cross-sectional moment of inertia of femoral shaft (cm^4^) |
|  | FS-Z | Section modulus of femoral shaft (cm^3^) |
|  | IT-CT | Intertrocanteric average cortical thickness (cm) |
|  | IT-BR | Intertrocanteric buckling ratio (cm^3^) |
|  | IT-CSA | Intertrocanteric Cross-sectional area (cm^2^) |
|  | IT-CSMI | Intertrocanteric Cross-sectional moment of inertia (cm^4^) |
|  | IT-Z | Intertrocanteric section modulus (cm^3^) |
|  | NN-CT | Average cortical thickness of narrow neck (cm) |
|  | NN-BR | Buckling ratio of narrow neck (cm^3^) |
|  | NN-CSA | Cross-sectional area of narrow neck (cm^2^) |
|  | NN-CSMI | Cross-sectional moment of inertia of narrow neck (cm^4^) |
|  | NN-Z | Section modulus of narrow neck (cm^3^) |

*HAL (mm):* the distance from pelvic rim to outer margin of greater trochanter along neck axis. *NSA (degrees):* angle between derived axes of neck and shaft. *FS:* the femoral shaft , *CT (cm):* estimate of mean cortical thickness. *BR (cm^3^):* Relative thickness of the cortex as an estimate of cortical stability in buckling. *CSA (cm^2^):* equivalent to the amount of (cortical equivalent) bone surface area in the cross-section after excluding all trabecular and soft tissue spaces. *CSMI (cm^4^):* for bending in the image plane from bone mass profile integral. Index of structural rigidity; reflects distribution of mass about the center of a structural element. *Z (cm^3^):* Indicator of bending strength for maximum bending stress in the image plane. *IT:* Intertrochanteric. *NN*: Narrow neck. [30]
