# Supplementary material for: Genetic Contribution of Femoral Neck Bone Geometry to the Risk of Developing Osteoporosis: A Family-Based Study
Source: PLoS One. 2016 May 10;11(5):e0154833. doi: 10.1371/journal.pone.0154833 (PMC4862643; doi:10.1371/journal.pone.0154833)
Supplement: S2 Table — (DOC) [file pone.0154833.s002.doc]

Table 2. Description of the general characteristics of the patients in each of the three categorical phenotypes.

| **Categorical phenotype** | **N** | **Median Age** | **M:F ratio** | **Median BMI** | **Median Age menopause** | **Median T-score** | | |
| --- | --- | --- | --- | --- | --- | --- | --- | --- |
|  |  |  |  |  |  | **Femoral neck BMD** | **Hip BMD** | **Total spine BMD** |
| **Affected 1** | 70 | 61.5 | 0.49 | 25.74 | 49 | -2.2 | -1.35 | -2.85 |
| **Affected 2** | 24 | 65 | 0.41 | 24.97 | 50 | -1.65 | -0.80 | -1.60 |
| **Affected 3** | 206 | 49.5 | 0.75 | 24.91 | 49 | -1.50 | -0.75 | -1.80 |

Affected 1: osteoporotic patients; Affected 2: patients with osteoporotic fractures; Affected 3: patients with low bone mass; N: number size; M: male; F: female.
