# Supplementary material for: Genetic Contribution of Femoral Neck Bone Geometry to the Risk of Developing Osteoporosis: A Family-Based Study
Source: PLoS One. 2016 May 10;11(5):e0154833. doi: 10.1371/journal.pone.0154833 (PMC4862643; doi:10.1371/journal.pone.0154833)
Supplement: S3 Table — (DOC) [file pone.0154833.s003.doc]

**Table 3. Heritability of the phenotypes in the GAO Project.**

|  | **Trait** | **h^2^ (h^2^ s)** | **P value** |
| --- | --- | --- | --- |
| **Structural traits** | HAL | 0.377 (0.081) | 2.95 × 10^-10^ |
|  | NSA | 0.456 (0.110) | 3.26 × 10^-08^ |
| **Strength properties** | FS-CT | 0.394 (0.098) | 2.5 × 10^-06^ |
|  | FS-BR | 0.454 (0.090) | 1.22 × 10^-09^ |
|  | FS-CSA | 0.252 (0.094) | 8.84 × 10^-04^ |
|  | FS-CSMI | 0.430 (0.098) | 2 × 10^-07^ |
|  | FS-Z | 0.354 (0.098) | 9.6 × 10^-06^ |
|  | IT-CT | 0.492 (0.096) | 1.77 × 10^-08^ |
|  | IT-BR | 0.493 (0.083) | 5.61 × 10^-12^ |
|  | IT-CSA | 0.353 (0.098) | 1.5 × 10^-05^ |
|  | IT-CSMI | 0.314 (0.098) | 5.72 × 10^-05^ |
|  | IT-Z | 0.328 (0.104) | 1.25 × 10^-04^ |
|  | NN-CT | 0.472 (0.104) | 5 × 10^-07^ |
|  | NN-BR | 0.586 (0.087) | 1.46 × 10^-12^ |
|  | NN-CSA | 0.308 (0.099) | 2.44 × 10^-04^ |
|  | NN-CSMI | 0.386 (0.097) | 3.9 × 10^-06^ |
|  | NN-Z | 0.277 (0.094) | 4.6 × 10^-04^ |

*(h^2^ s): h^2^ standard error.* See table 1 for acronym descriptions.
