# Supplementary material for: Genetic Contribution of Femoral Neck Bone Geometry to the Risk of Developing Osteoporosis: A Family-Based Study
Source: PLoS One. 2016 May 10;11(5):e0154833. doi: 10.1371/journal.pone.0154833 (PMC4862643; doi:10.1371/journal.pone.0154833)
Supplement: S4 Table — (DOC) [file pone.0154833.s004.doc]

**Table 4. Regression coefficients for statistically significant covariate effects.**

|  | **Trait** | **Age** | **Female gender** | **BMI** | **Menopause age** | **Var. expl** |
| --- | --- | --- | --- | --- | --- | --- |
| **Structural traits** | HAL | 0.216 | -1.5.9 |  |  | 0.558 |
|  | NSA |  | -1.801 |  | 0.052 | 0.0754 |
| **Strength properties** | FS-CT |  | -0.07 | 0.009 | -0.002 | 0.3557 |
|  | FS-BR | 0.008 |  | -0.024 | 0.007 | 0.196 |
|  | FS-CSA | 0.016 | -1.077 | 0.081 | -0.008 | 0.6889 |
|  | FS-CSMI | 0.027 | -1.662 | 0.1 |  | 0.697 |
|  | FS-Z | 0.014 | -0.823 | 0.054 |  | 0.7309 |
|  | IT-CT |  | -0.035 | 0.004 | -0.001 | 0.2362 |
|  | IT-BR | 0.047 |  | -0.065 | 0.016 | 0.2908 |
|  | IT-CSA | 0.01 | -1.057 | 0.074 | -0.011 | 0.4811 |
|  | IT-CSMI | 0.093 | -5.499 | 0.298 |  | 0.6109 |
|  | IT-Z | 0.02 | -1.328 | 0.089 |  | 0.5594 |
|  | NN-CT | 0 |  | 0.002 | 0 | 0.199 |
|  | NN-BR | 0.074 | -1.141 | -0.08 |  | 0.2804 |
|  | NN-CSA |  | -0.533 | 0.044 | -0.006 | 0.4325 |
|  | NN-CSMI | 0.015 | -1.399 | 0.063 |  | 0.5732 |
|  | NN-Z | 0.005 | -0.524 | 0.026 | -0.004 | 0.508 |

Only significant p values are shown (p<0.05). Empty spaces means that the effect was not significant (p≥ 0.05)

var.expl: variance explained by adjusted covariates; -: not analyzable. See table 1 for acronym descriptions.
