# Supplementary material for: Genetic Contribution of Femoral Neck Bone Geometry to the Risk of Developing Osteoporosis: A Family-Based Study
Source: PLoS One. 2016 May 10;11(5):e0154833. doi: 10.1371/journal.pone.0154833 (PMC4862643; doi:10.1371/journal.pone.0154833)
Supplement: S5 Table — (DOC) [file pone.0154833.s005.doc]

**Table 5. Genetic and environmental correlation of intermediate phenotypes based on femoral geometry parameters with three different osteoporotic phenotypes.**

|  | **Trait** | **Affected1 n=66** | | **Affected2 n=24** | | **Affected3 n=216** | |
| --- | --- | --- | --- | --- | --- | --- | --- |
|  |  | **ρ_G_** | **ρ_E_** | **ρ_G_** | **ρ_E_** | **ρ_G_** | **ρ_E_** |
| **Structural traits** | **HAL** | -0.208 | 0.003 | 0.234 | -0.098 | 0.010 | 0.241 |
|  | **NSA** | -0.122 | -0.055 | -0.023 | -0.302 | 0.154 | -0.267 |
| **Strength properties** | **FS-CT** | -0.543 | -0.354 | -0.484 | -0.139 | -0.781* | 0.149 |
|  | **FS-BR** | 0.488 | 0.460 | 0.770* | -0.011 | 0.644 | 0.179 |
|  | **FS-CSA** | -0.429 | -0.082 | 0.473 | -0.230 | -0.762* | 0.320 |
|  | **FS-CSMI** | -0.140 | 0.163 | 0.896* | -0.259 | -0.216 | 0.379 |
|  | **FS-Z** | -0.167 | 0.108 | 0.957* | -0.271 | -0.281 | 0.396 |
|  | **IT-CT** | -0.758* | -0.452 | -0.515 | -0.024 | -0.954* | -0.107 |
|  | **IT-BR** | 0.824* | 0.509 | 1.000* | -0.027 | 0.775* | 0.732* |
|  | **IT-CSA** | -0.641 | -0.161 | -0.076 | -0.122 | -0.857* | 0.160 |
|  | **IT-CSMI** | -0.537 | 0.110 | 0.395 | -0.091 | -0.669 | 0.340 |
|  | **IT-Z** | -0.634 | 0.058 | 0.244 | -0.116 | -0.751* | 0.275 |
|  | **NN-CT** | -0.497 | -0.693 | -0.178 | -0.390 | -0.617 | -0.440 |
|  | **NN-BR** | 0.458 | 0.656 | 0.557 | 0.161 | 0.505 | 0.847* |
|  | **NN-CSA** | -0.526 | -0.360 | 0.282 | -0.372 | -0.743* | -0.024 |
|  | **NN-CSMI** | -0.701* | 0.022 | 0.622 | -0.180 | -0.748* | 0.250 |
|  | **NN-Z** | -0.656 | -0.124 | 0.464 | -0.253 | -0.694 | 0.089 |

**: results with relevant genetic correlations.*

ρ_G_: genetic contribution; ρ_E_: environmental contribution. See text for the definition on Affected1 to 3. See table 1 for acronym descriptions.
